# Evolution, hibernation, and inactivation of voltage-gated Na channels

**DOI:** 10.1085/jgp.202513923

**Published:** 2026-07-07

**Authors:** John S. Willis

**Affiliations:** 1 Department of Cellular Biology, University of Georgia, Athens, GA, USA

## Abstract

At 6°C, when atria of most mammals cannot be activated, isolated atria of 13-lined ground squirrels, a model hibernator, have intracellular action potentials (AP) that are enlarged in duration and overshoot. Avoidance or delay of inactivation was postulated as playing a role in the retention and enhancement of the AP. In a published genomic study, the TTX-sensitive voltage-gated Na channel Nav1.2 was identified as being “more rapidly evolving.” In published sequences of this channel in five species of hibernators, a methionine replaces leucine in the sequence, NQATL, of transmembrane segment six of domain one (DIS6), but not in any of the 10 species of nonhibernating placental mammals. Among other TTX-sensitive channels, Nav1.1 possesses the same mutation in 8 of 9 hibernators as does Nav1.4 in bats and Nav1.3 in groundhogs, but those isoforms do not possess the mutation in any nonhibernator. All four channels occur in the heart muscle. While far less abundant than Nav1.5, they may supplement it during physiological stress. In a model of Nav1.4A, methionine replacing leucine in NEATL (homolog of NQATL) was found to form two hydrogen bonds with the asparagine of NEATL. Leucine formed none. That asparagine is highly conserved in all members of the Nav1 family and mpdulates both fast and slow inactivation. Hypothesis: At temperatures approaching 0°C, greater stickiness of methionine enhances the nearby arginine’s modulation of fast and slow inactivation, thus helping to sustain excitability in hearts of hibernators that possess the M-for-L mutation.

## Teaser

Hearts of hibernating mammals continue to beat at near 0°C thanks to continued excitability. Several members of a minor group of voltage-gated Na channels in the heart are found to contain a mutation unique to hibernators. Molecular details of this mutation may help to explain the continued activity of the heart in the cold.

## Introduction

In 2023, the journal *Science* devoted an entire section, called “Zoonomia,” to whole genomes of 240 diverse species of placental mammals. In that section, Christmas et al. (2023) described studies on placental mammals with special metabolic adaptations—or traits—such as aquatic life, hibernation, and flight. They undertook to identify “rapidly evolving” trait-specific variations by comparing protein-coding sequences of both closely related and less related species with and without the specific trait. Low variation signified conservation, greater variation possible selection or “evolution.” For hibernation, they selected 22 hibernating species capable of sustaining a body temperature below 18°C for >24 h and compared them with 154 species that maintain high body temperatures at all times.

They found 11 genes evolving faster among the hibernating species, and among these was SCN2A, which codes for Nav1.2A, a member of the family of voltage-gated (v.g.) Na channels. This result was of particular interest to me since my very first published paper (Marshall and Willis, 1962) dealt with transmembrane action potentials (AP) of 13-lined ground squirrels, a quintessential hibernator. It had been well established previously that whole hearts of hibernators perfused in vitro continue to beat at near 0°C temperatures, whereas those of mammals incapable of hibernating stopped below 10°C (Lyman and Blinks, 1959). Marshall (1957) had also found that in isolated atria of rabbits, resting potentials fell linearly with cooling and ceased to conduct AP below 11°C.

In contrast, we found in isolated atria from hibernating ground squirrels that while resting potential declined at 6°C, the height of AP increased by 10 mV from +80 to +90, thanks to a 12 mV increase in overshoot. In those days, before the wide use of tape recorders, events on the oscilloscope were captured by photographs of the screen, and later the image was projected onto a horizontal surface, and parameters were measured by hand. I well recall that switching from 33°C recordings to 6°C ones, I had to lower the projector several inches to get on the screen the whole of what I dubbed “giant AP.” (Duration at half recovery increased almost fivefold from 12 msec at 33°C to 57 msec at 6°C; heart rate declined from 258/s to 21.)

In our published paper, we speculated that the greater overshoot could have been due to delayed inactivation of the inward Na current that would allow the membrane potential to more closely approach the E_Na._ We made no mention of the then-hypothetical v.g. Na channel. Now, 60 years later, with actual v.g. Na channels having been identified, sequenced, and described structurally and shown by physiologically blind genomic analysis that one of them was a player in low temperature adaptation, I wondered whether by exploring the available data for this molecule I could gain answers to what was going on in our long-ago recordings.

Two caveats will need to be considered in weighing the importance of Nav1.2 in relation to events in cardiac muscle. First, the principal v.g. channel in the heart muscle is Nav1.5. Nav1.2 is one of several channels present at lower levels of expression in heart muscle that are, unlike Nav1.5, blocked by low concentrations of the drug tetrodotoxin (TTX). The most abundant of these is Nav1.4 (Haufe et al., 2005), usually associated with skeletal muscle. Four others (Nav1.1, 1.2, 1.3, and 1.6) are principally associated with brain and peripheral neurons. Secondly, other currents could have contributed to the old observations, in particular those mediated by v.g. Ca channels that are present in the pacemaker and conducting tissue of the atria (Striessnig et al., 2014).

## Materials and methods

### Selection of species

Lyman and Chatfield (1955) defined “deep hibernation” as the state small mammals achieve when, faced with a cold environment, they voluntarily lower their body temperature to a minimum difference as little as 0.5°C above ambient, down to as low as 0°C, and periodically rewarm spontaneously without an external source of heat. I have selected species that have been well studied and meet this stringent criterion, and for which I could find a sequence in the NCBI database for the TTX-sensitive isoform of Nav1 documented in the heart. The accession numbers are provided in Table 1.

**Table 1.** NCBI Accession numbers for entries in Table 2

Common name	SCN1A	SCN2A	SCN3A	SCN4A	SCN8A
13-Lined ground squirrel	XP_077873649	KAG3274613	N.A.	XP_021584085	XP_077870796
Arctic ground squirrel	XP_077654796	XP_026266882	XM_026411111	KAM4822770	KAM4840914
Yellow-bellied marmot	XP_027801819	XP_027801838	N.A.	XP_027802102	XP_027805589
Groundhog	XP_058436131	XP_058436122	XP_046303543	XP_046283698	XP_058432357
European marmot	XP_015334753	XP_015334460	N.A.	XP_015360302	XP_048658711
Golden hamster	XP_040601424	*XP_021088572*	N.A.	XP_005070068	XP_040609720
European hedgehog	XP_060033761	XP_060033766	N.A.	XP_060058537	XP_060051330
Little brown bat	XP_023601115	XP_036176358	XP_023601169	XP_023612521	XP_023615422
Big brown bat	XP_054578784	XP_054578791	N.A.	XP_008147646	XP_054575045
Beaver	N.A.	XP_020007198	XP_073927097	XP_073901513	XP_073939522
Guinea pig	XP_063115272	XM_063259190	XP_023416928	XP_063098664	XP_063110857
Deermouse	XP_076425700	XP_052581787	XP_076425693	XP_015849809	XP_076412978
Norway rat	NP_110502	NP_036779	NP_001424699	NP_037310.2	XP_063119207
Eastern gray squirrel	XP_047400384	XP_047399883	XP_047400815	XP_047400804	XP_047404261
Rabbit	N.A.	XP_008256916	XP_069926841	XP_002719526	XP_051701456
Dog	XP_038302870	XP_013966299	XP_038302855	XP_038531929	XP_038294064
Shrew	XP_054975660	XP_054978862	XP_004601169	XP_054987535	XP_055976251
Elephant	XP_064143113	XP_003405869	N.A.	XP_064126987	XP_023412327
Horse	XP_070098085.1	XP_023478830.1	XP_070098078	NP_001075230	XP_070127551
Human	P35498	M94055	AAK00219.1	P35499	AAD15789

This list differs from that of Christmas et al. (2023) in several respects. They chose 18°C as their standard for low-maintained body temperature, whereas standard species used in physiological research (marmots, hedgehogs, ground squirrels, and hamsters) sustain body temperatures well below 10°C. Consequently, their list contains two species not generally recognized as deep hibernators, the polar bear and the Indochinese shrew. The list of Christmas et al. (2023) also omits two species that are capable of deep hibernation, Chinese hamsters and the European hedgehog. The latter is a classic model for hibernation studies by European physiologists (e.g., Johansson and Senturia, 1972). (The shrew and hamster, however, are next to each other in their table listing species. Conceivably, the names may have been switched at some late stage of publication, without affecting their appropriate placement in the data collection.)

For nonhibernating species, I chose a few widely separated phylogenetically from the hibernators (elephant, human, dog, rabbit, and horse) and others more closely related (eastern gray squirrel, rodents generally, the insectivore shrew to compare with hedgehogs). I was unable to find a sequence for any nonhibernating microchiropteran to compare with the big and the little brown bat.

### Choice of model protein

The RCSB PDB designation for human Nav1.4 used is 6AGF. The version of viewer used is Glaxo Smith Kline, DeepView, Swiss-PDB Viewer, 1995–2001 by Nicolas Guex and colleagues.

## Results

### Sequence comparisons of Nav1.2A

Much of the progress in understanding v.g. channels has been in identifying the molecular identity of the operative components, such as the pore formed by the six transmembrane segments (S6) of each of the four domains (DI–DIV). Of particular relevance for fast inactivation are the fourth transmembrane segments of domains III and IV (DIIIS4 and DIVS4) containing the voltage-sensitive (VS) region, and the sequence of isoleucine, phenylalanine, methionine (IFM) in the cytoplasmic linker between DIII and DIV.

The sequences of all the Nav1 family of channels are highly conserved both between species and between isoforms, so that it was not surprising that a quick examination of Nav1.2 A of the 13-lined ground squirrel and rat revealed no difference whatever in the VS (or indeed the whole S4) of DIV nor in the region of the DIII–DIV linker within many residues either side of IFM.

I undertook to see if I could find *any* differences in sequences of Nav1.2A between the 13-lined ground squirrel and rat, as well as those of two other standard nonhibernators, the guinea pig and human. I found only six amino acids that are different in the ground squirrel from all three of the other species. To determine if these differences were just specific to the ground squirrel, I expanded the sample to nine well-studied species of hibernator and 11 species of familiar nonhibernating mammals and looked for cases of a variant that was (1) found only in hibernators; (2) found in at least two species not closely related phylogenetically (like ground squirrels and marmots); (3) the “normal” amino acid is highly conserved in all nonhibernators. Only one variant amino acid met these criteria: The leucine (L) present in all species of nonhibernators in a sequence, NQATL, near the end of transmembrane segment six of domain one (DIS6) is replaced by a methionine (M) in 5 species of hibernators (Table 2).

**Table 2.** Prevalence of methionine-for-leucine substitution in TTX-sensitive v.g. Na channels of the hearts of hibernators

Common name	Technical name	NQATX.., X = ?					Hibernator?
		SCN1A Nav1.1A	SCN2A Nav1.2A	SCN3A Nav1.3A	SCN8A Nav1.6A	SCN4A Nav1.4A	
13-Lined ground squirrel	*Ictidomys tridecemlineatus*	M	M	______	L	L	Yes
Arctic ground squirrel	*Urocitellus parryii*	M	M	L	L	L	Yes
Yellow-bellied marmot	*Marmota flaviventris*	M	M	______	L	L	Yes
Woodchuck or groundhog	*Marmota monax*	M	L	M	L	L	Yes
European marmot	*Marmota marmota*	M	L	______	L	L	Yes
Golden hamster	*Mesocricetus auratus*	L	L	______	L	L	Yes
European hedgehog	*Erinaceus europeus*	M	L	L	L	L	Yes
Little brown bat	*Myotis lucifugus or Myotis*	M	M	L	L	M	Yes
Big brown bat	*Eptesicus fuscus*	M	M	______	L	M	Yes
Beaver	*Castor canadensis*	_____	L	L	L	L	No
Guinea pig	*Cavia porcellus*	L	L	______	L	L	No
Deermouse	*Peromyscus* spp.	L	L	______	L	L	No
Norway rat	*Rattus norvegicus*	L	L	L	L	L	No
E gray squirrel	*Sciurus carolinensis*	L	L	______	L	L	No
Rabbit	*Oryctolagus cuniculus*	______	L	______	L	L	No
Dog	*Canis lupus familiaris*	L	L	______	L	L	No
Shrew	*Sorex araneus*	L	L	______	L	L	No
Elephant	*Loxodonta africanus*	L	L	______	L	L	No
Horse	*Equus caballus*	L	L	L	L	L	No
Human	*Homo sapiens*	L	L	______	L	L	No

The segment in which an M-for-L mutation is found is in the sixth transmembrane segment of domain one (DIS6) and begins NQAT in most Nav1s. (In Nav1.4, glutamate replaces glutamine, thus NEAT.) Criteria for the selection of hibernators are provided in Materials and methods. Briefly, nonhibernators resist lowering of body temperature below 30°C in a cold environment by increasing heat production and insulation. Hibernators listed in the table in low ambient temperature and under appropriate circumstances, specific to the species, allow or even promote a decrease in core temperature to below 10°C and to as low as 0°C and regulate temperature and respiration at that low temperature for days or weeks. Dashed lines indicate that no sequence was available in the NCBI database. In the headings of each column, gene names are shown above protein identities.

I then examined this location in the other TTX-sensitive Nav1’s of the heart, and found that Nav1.1 shows the same mutation in all the hibernators except the hamster (Table 2). In Nav1.4, methionine replaces leucine in the bats. For Nav1.3, sequences were unavailable for half the hibernators, but for those that were available, methionine replaces leucine in the groundhog. In Nav1.6, the leucine is conserved in all 20 species.

For Nav1.5, the major v.g Na channel of the heart, the corresponding sequence is NQATI in all 20 species, and no variant in Nav1.5A meets the criteria stated above that allowed the identification of the M-for-L replacement in TTX-sensitive channels as being unique to hibernators.

### Position and consequences of M-for-L mutation

In the past decade the long-held and widely accepted hypothesis that the IFM in the DIII–DIV linker moves to block the cytoplasmic mouth of the pore during inactivation has been under reconsideration (Liu et al., 2025; Hull and Lampert, 2025) because structural studies have shown that it is too far from the pore’s mouth to accomplish that act and that during inactivation it docks in a groove in the wall of S6 segments that form the wall of the pore but on the side of the wall opposite the pore. Nav1.4A (SCN4A) has been a prominent example both for EM studies of structure and for expression studies of changes in activity resulting from site-directed mutagenesis (Pan et al., 2018). In those structural studies, the molecule appears to be in a state of inactivation with the S4 segments shifted upward and the IFM docked. Therefore, I chose to use the PDB model of Nav1.4A that had been employed for those studies to investigate the possible impact of converting L458 to M458 in human Nav1.4.

**Figure 1.**
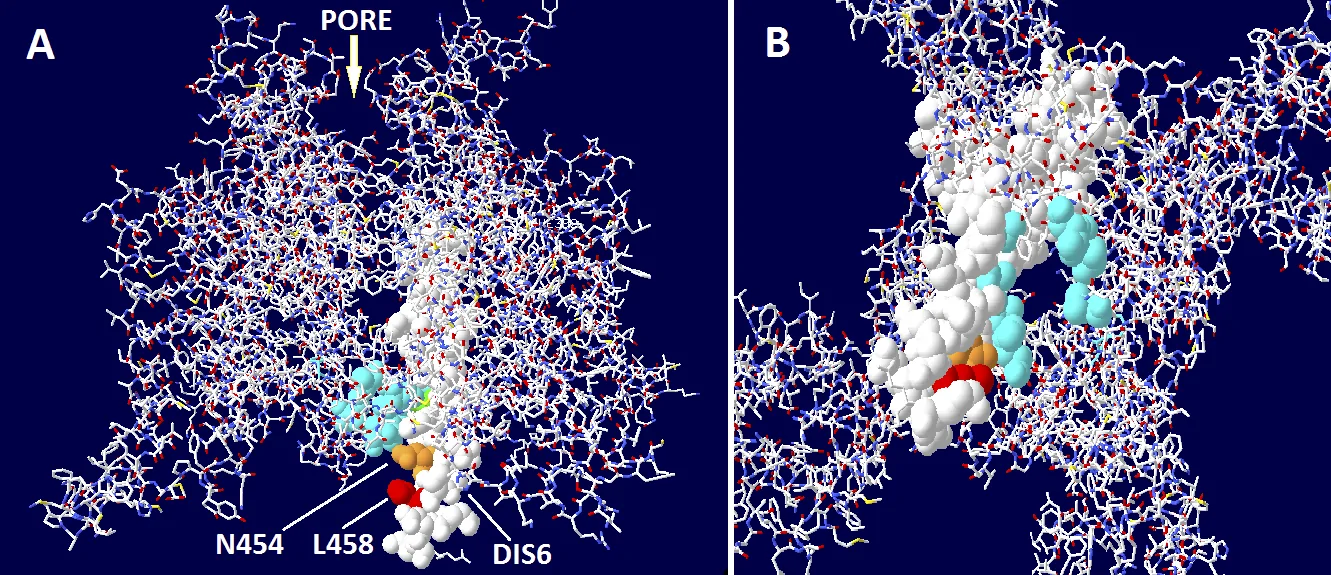
**Human Nav1.4A. (A)** (PDB model 6AGF). Side view, cytoplasmic side down. White spheres, 3-D depiction of atoms of molecules of DIS6 and part of the S5–S6 linker that form one quarter of the wall of the channel. The other three S6 segments and linkers that complete the channel are retained as sticks to allow the view of the location of N454 (orange spheres) of the sequence NEATL and of L458 (red spheres) of the same sequence. Blue spheres, eight residues, two from each S6 segment, form a double ring structure at the narrowest part of the pore at the cytoplasmic end. **(B)** Same model as shown in A, flipped upward and magnified to show a vertical view from the end of DIS6 and into the channel from the cytoplasmic side.

The sequence in which the variance occurs, NEAT(L/M) in Nav1.4, is about six residues from the end of the DIS6 helix (Fig. 1). The asparagine (N) of the sequence is highly conserved in all Nav1’s, and it is the only one in DIS6 and is sometimes referred to as DIS6N. In most 3-D molecular models of Nav1’s, DIS6N appears to be at the last position in the wall of the pore, so that the remaining residues of the DIS6 helix are in the cytoplasm, not in the pore (Fig. 1 A). Although the L-or-M residue is separated from DIS6N by three places, it lies adjacent to DIS6N, thanks to the turn of the helical coil. Seven residues into the membrane from DIS6N is a sequence, LAVVA, with two residues, the leucine and the alanine, after valine (colored blue in Fig. 1), that are one of four pairs of amino acids that constitute the narrowest stretch of the pore and are variously involved in inactivation (Liu et al., 2025). The asparagine, DIS6N (or N454 in the model, Fig.1), has itself also been found to modulate fast and slow inactivation (Wang and Wang, 1997).

**Figure 2.**
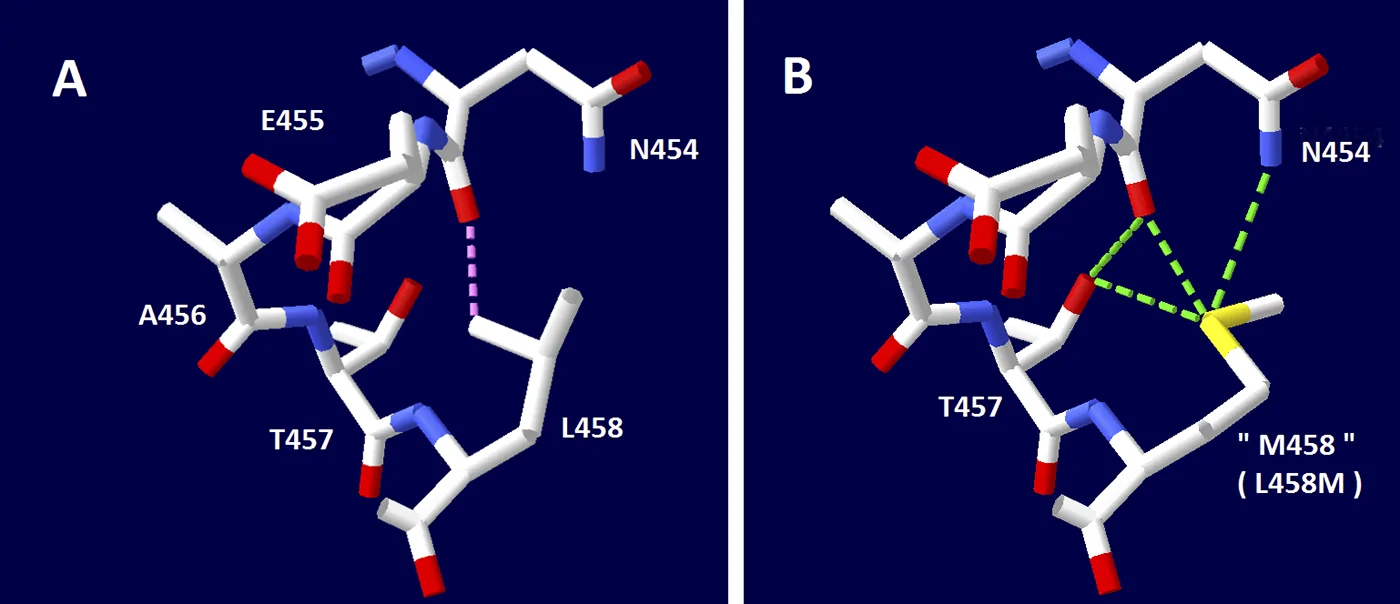
**Detail of NEATL in DIS6. (A)** Detail of NEATL sequence in DIS6. Pink dotted line, potential clash. **(B)** Same, with highly conserved leucine replaced by methionine, as it is in this isoform in bats and in Nav1.1 and 1.2 in bats and other species of placental mammalian hibernators (Table 2). Green dotted lines, potential hydrogen bonds.

Fig. 2 A shows the detail of the sequence, NEATL of Nav1.4, within the conformation of the model illustrated in Fig. 1. Using the “rotation” tool of the Swiss-PDB Viewer and manipulating both the leucine (L458) and the asparagine (N454), I could find no combination of positions of side arms that would yield a hydrogen bond between the two. There were, however, numerous opportunities for clashes, one of which is illustrated in Fig 2 A.

When I substituted methionine for leucine in NQATL (i.e., L458M), using the “mutation” tool in the Swiss-PDB Viewer and then applying the rotation tool, I found the methionine capable of forming two hydrogen bonds with the asparagine (Fig. 2 B).

When several bonds form simultaneously, as shown in Fig 2 B, they create a potentially more stable attraction between the two molecules. Captured in that figure is a rare but apparently possible combination with three levels of interaction: First, there is a hydrogen bond between methionine’s sulfur atom and the carboxyl oxygen of the asparagine, then there is another hydrogen bond between the sulfur atom and the side chain nitrogen (or, alternatively, oxygen) of asparagine. The first bond stabilizes the second. Finally, the sulfur atom of methionine can also form a hydrogen bond with the side chain of the neighboring threonine in NEATL, as can the asparagine, creating a complex. At normal mammalian body temperatures, these connections would be fleeting, given the random movements and positions of side chains. But at temperatures approaching 0°C, they would become more lingering and possibly more consequential.

## Discussion

This study began as an attempt to see if a finding in the recent genomic study of Christmas et al. (2023) could help explain an old observation of Marshall and Willis (1962). That “finding” was higher variability (interpreted as more rapid evolution) of the v.-g. Na channel Nav1.2 in species of animals that hibernate, and the “old observation” was increased overshoot at 6°C in atrial cells of ground squirrels, a quintessential hibernator.

The results, above, show that multispecies comparison of the amino acid sequence of Nav1.2 yields only a single amino acid variance that is found solely in hibernators (Table 2), the M-for-L substitution in the DIS6 sequence, NQATL. That same mutation also occurs in NQATL of Nav1.1, where it is even more pervasive among hibernators than Nav1.2 and in Nav1.3 in the genus *Marmota* (groundhog, Table 2, sequences for the other marmots were not available). It also occurs in NEATL of Nav1.4 in bats, but it does not occur in any of the isoforms of any of the nonhibernating mammals examined. Thus, among the hibernators examined in this study for which sequences were available, every one of them except the hamster possesses at least two TTX-sensitive isoforms of Nav1 with the mutation (bats have three!). The likelihood that this mutation is relevant to cold resistance in excitable cells of hibernators seems hard to deny; what remains is whether that relevance applies to their hearts.

### TTX-resistant Nav1 isoforms in the heart

The isoform Nav1.5 is the principal v.g (voltage-gated) Na channel in cardiac muscle, and it has a low sensitivity to TTX. Effects of low concentrations (5–25 nM) of TTX on the cardiac AP have been documented since 1979 (Coraboeuf et al., 1979) and the presence of mRNA for TTX-sensitive v.-g. channels has been found in the heart since 1989 (Sills et al., 1989). Haufe et al. (2005) quantified these effects and also examined the localization of specific isoforms in developing and adult mouse ventricular cardiomyocytes. Using quantitative PCR, they found that Nav1.4 was the most abundant and Nav1.6 the least abundant of the mRNAs (cDNAs) of the TTX-sensitive isoforms and that Nav1.2 was significantly more abundant than Nav1.1. Using patch clamp of whole myocytes, they differentiated between current through Nav1.5 and that of the TTX-sensitive channels by using two test voltages, −50 mV to activate Nav1.5 and −5 mV to encompass the TTX sensitive channels. From the results of these measurements, they were able to compute that 8% of the Na current during activation was carried through the TTX pathways. Their immunolocalization studies showed that Nav1.5 was localized in the intercalated disks and over the Z bands of myocytes, as were both Nav1.1 and 1.2. Images for Nav1.3 were mostly diffuse, but in some cases were punctate over Z lines. Those for Nav1.6 were weaker but also associated with Z bands. Other studies have shown that distribution within the heart of TTX-sensitive channels varies: Nav1.1 is more prominent in the sinoatrial tissue (Maier et al., 2003) and in the atrioventricular junction (Yoo et al., 2006).

In human atrial myocytes, Kaufmann et al. (2013) found that Nav1.5 accounted for 88% of the immunochemically identifiable sites, and they were located over the Z lines, as was Nav1.4. Nav1.2 was located primarily in the intercalated discs but also, more faintly, over the Z lines. Staining for the remaining three TTX-sensitive channels was punctate and scattered over the cell surface. In the same study, whole cell patch clamp of myocytes isolated from the atrial samples and tested at room temperature showed that up to 27% of the current was TTX-sensitive. Separately, in tissue samples stimulated electrically and with autonomic endings blocked chemically, TTX impaired the development of maximal contraction.

While such studies demonstrate that TTX-sensitive channels are present throughout the heart and are involved collectively in some way with normal capacity for function, they do not demonstrate the need for any particular isoform. Clues on that point have depended largely upon reports that are byproducts of interest in epilepsy. For example, Tzialla et al. (2022) found sustained tachycardic arrhythmia in addition to neurological symptoms in a newborn with a de novo pathogenic mutation in Nav1.2A. Varghese et al. (2011. *Circ. Res.* Abstract AP081. https://doi.org/10.1161/res.109.suppl_1.AP081) found various structural abnormalities and diminished performance in the hearts of mice with a knock-in of an epileptogenic mutation of Nav1.1. In another mouse model with a mutation in Nav1.1 causing Dravet’s syndrome epilepsy, complex electrical changes in whole in vivo hearts and in isolated cardiomyocytes signified hyperexcitability contributing to—or causing—sudden unexplained death in epilepsy (Auerbach et al., 2013).

### Delay of fast inactivation and avoidance of slow inactivation in the cold heart

In our 1962 study of AP in ground squirrel atria, Marshall and I raised the possibility that delay in fast inactivation was a possible cause of the increased overshoot at low temperature. In the same cells, membrane potential was slightly but significantly reduced from that at higher temperatures, a fact that might also implicate avoidance of slow inactivation as a contributor to the persistence and enhancement of the AP in the cold.

In that 1962 study, Marshall and I used a floating microelectrode suspended on a folded filament of foil. At high temperature with rapid heart rate, the movement aided penetration, but at low temperature with infrequent contraction, obtaining a stable, reliable penetration was more difficult. Consequently, the only resting potentials that we could be sure of were those in which we captured a strong AP. So it is very possible that there are regions in the atria with more depressed resting potentials with conduction block. An earlier study of EKG in hibernating ground squirrels reported some bundle branch block (Nardone, 1955). In such a situation, an elevated AP, constituting a stronger current sink, could allow sufficient spread to ensure transmission and contraction by a sort of saltatory conduction.

The situation in ground squirrel hearts, or those of any deep hibernator at temperatures near 0°C, may be only a special case of the competition between delay of fast inactivation, allowing greater depolarization, and avoidance of slow inactivation shutting down conduction. In her studies of rabbit atria, Marshall (1957) saw a progressive decline in resting membrane potential and in overshoot even with mild cooling, whereas with steady resting membrane potentials down to 15°C in guinea pig atria, Illanes et al. (1970) saw a 4 mV increase in overshoot between 35 and 25°C and a further 10 mV rise between 25 and 15°C.

Lowering temperature tends to slow down most processes, but those related to diffusive or random intramolecular movements tend to be proportional to change in absolute temperature for which zero kelvin (0°K) is at −273°C, so that the 27°C difference under discussion here would represent a 9% decrease (33°C = 306°K and 27/306 = 0.09) and would account for a relatively small effect. However, fast inactivation involves the prior movement outward of DIIIS4 and DIVS4 that entrains the transfer of IFM to its binding site (Jiang et al., 2020). The outward movement of all four of the S4’s requires their voltage-sensing lysines and arginines to break from ion pair attachments to negatively charged amino acids in neighboring S3 segments and punch through a hydrophilic zone (Jiang et al., 2020). Of particular relevance is the further slowing of the movement of DIVS4 that is necessary for inactivation, and the last S4 to move, the rate-limiting step. Resistance to the outward movement constitutes an energy barrier, and overcoming it requires a minimal energy of activation that by definition invokes a steep Arrhenius slope, i.e., exponential temperature dependence. Hence, the possibility of a relatively delayed onset of fast inactivation.

Resting membrane potentials are reduced by cooling in at least three ways: a smaller RT term in determining diffusion potential of K^+^, reduced positive outward Na current contributed by Na-K pump, and decline of K^+^ gradient owing to pump not keeping up with leak. For channels with a more negative threshold potential, these effects would lead to slow inactivation of their population. Ground squirrels and other hibernators are protected from the decline of the K gradient by better sustained pumping at low temperature (Willis, 1964; Kimzey and Willis, 1971), but are subject to the other two. Even for the slight decline in resting potential seen in ground squirrel atria at 6°C, slow inactivation may still be a risk given the exceedingly long times involved, 3 s between beats and 1–2 wk of sustained temperature near 0°C.

Hence, a little extra protection against slow inactivation might be useful for survival. TTX-sensitive channels may fit this need. As noted above in the whole-cell patch-clamp studies of Haufe et al. (2005) and also those of Kaufmann et al. (2013), they activate at a less negative potential than Nav1.5. This would seem to make them less vulnerable than Nav1.5 to sustained depolarization of resting potential. If TTX-sensitive channels are supplemental or accessory to Nav1.5, this could be one of their roles: to come into play when Nav1.5 activity is threatened by metabolic challenge. Kaufmann et al. (2013) stated the idea this way: “The presence of TTX-sensitive sodium channels with the positive voltage dependence of gating…suggests additional roles for TTX-sensitive channels. In addition to their function in conducting the AP within the cell and from cell to cell, they may play a safety function in diseased myocardium where cells have a less hyperpolarized resting membrane potential with the consequence that the predominant Nav1.5 channels may be unavailable for activation.” For our purposes here, we can substitute “cold” for the word “diseased.” Accordingly, TTX channels should already be on the job at room temperature, so we should expect that the TTX-sensitive component of inward Na current measured by Kaufman et al. would have disappeared entirely at 37°C, as was indeed the case in another study of a TTX-sensitive Na current in human atria (Poulet et al., 2015).

### Another possible component of giant AP: Ca currents

The preceding argument has been that the TTX-sensitive Nav1s can supplement failing Nav1.5, maintaining and even increasing the size of the AP, partly because they have a less negative threshold potential and are presumably less susceptible to slow inactivation due to depolarization. On this point, the same could also be said of the Ca current provided by Cav1.2, an L-type v.g. Ca channel in the atria. In conducting and contracting atrial fibers, it is activated at a less negative membrane potential than Nav1.5 (Xu and Lipscombe, 2001). The other cardiac L-type Ca channel, Cav1.3, is a critical component in pacemaking in the sinoatrial node (Mangoni et al., 2003) and atrioventricular node (Striessnig et al., 2014) and behaves more like Nav1.5 in activating at a more negative potential and more quickly than Cav1.2 (Xu and Lipscombe, 2001). It is less strongly represented in conducting-contracting cells of the atrium (Mangoni et al., 2003). Both L-type Ca channels contribute to the AP even at normal mammalian body temperature and could do so in the cold. In the event of Na current through Nav1.5 failing at temperatures below 10°C, they could contribute to sustaining overshoot and are surely a likely cause of the greatly extended duration. But actually to increase the overshoot under those conditions would require that Cav1.2 has a mutation that delays its own voltage-dependent inactivation, allowing the membrane potential to more closely approach E_Ca_.

### Is there a role in the nervous system for M-for-L substitution in Nav1.1–1.3?

This narrative has been entirely centered on cardiac muscle and the contribution of v.g. Na channels that are usually identified with neurons. From an evolutionary perspective, the expectations of the heart and of the nervous system are quite different, nearly opposite: The presumed selective advantage of deep hibernation is survival of long starvation on body fat by extreme reduction in metabolic rate. To accomplish this, the entire muscle mass of the heart must continue to perform as an organized pump. On the other hand, operation of the nervous system, especially the brain, is very expensive, not only because of its high rate of respiratory metabolism, almost 50% of which is driven by ADP released by Na-K ATPase compensating for Na uptake resulting from electrical activity (Whittam, 1962), but also because of its sole reliance on carbohydrate stores, which are limited and reserved for the intensive effort of periodic arousal. Thus, it is imperative that its metabolism be strongly curtailed, not only by cold but, in the most favorable case, by any means that limits Na entry to cells (Hochachka, 1986). Viewed in this light, some form of temporary, reversible inactivation would be a blessing, not a curse, and the cellular mechanisms allowing these adjustments may be quite different between myocytes and neurons.

Accordingly, the brain is largely shut down during hibernation, with only a few systems still operating in subcortical areas involved with controlling respiration, thermoregulation, and response to external stimuli. In the peripheral nervous system, sensory neurons involved with pain and touch reception are still functioning, although possibly at reduced capacity (Hoffstaetter et al., 2018). Motor pathways, driving ongoing activities like respiration (e.g., phrenic nerve, South, 1961), vasomotor control, and arousal presumably remain at least minimally functional. (For reviews, see Sonntag and Arendt, 2019; Heller, 1979; Willis, 1979; Carey et al., 2003.)

The question remains of whether the analysis provided here for myocytes can be applied to those essential neurons that are still maintaining operation at the low body temperature of deep hibernation. Unfortunately, the foundation for answering this question does not yet exist. Two classic studies on isolated peripheral motor nerves of hamsters, stimulated by and recorded with extracellular electrodes, showed that they were able to conduct AP at temperatures below those causing failure in the same nerves of rats (Chatfield et al., 1948, on tibial nerve, and South, 1961, on phrenic nerve). There have, however, been no comparable studies with nerves of any of the other listed deep hibernators (Table 2), which, unlike the hamster (Table 2), carry the M-for-L mutation. Nor have there been any studies of transmembrane resting and AP of neurons of isolated nerves, measured at low temperature.

Lacking this essential information, we can still inquire what opportunities may exist for the M-for–L substitution to allow TTX-sensitive v.g. channels to maintain conduction of AP along axons of neurons of those species that have the mutation. Those opportunities are somewhat limited. Nav1.3 is not involved in axonal conduction in mature neurons of adults (Cheah et al., 2013). Nav1.2, while present along the axons of developing myelinated neurons, is replaced by Nav1.6 at the nodes of Ranvier in mature neurons of adults; it perdures in the axons of unmyelinated neurons (Boiko et al., 2001).

In the spinal cord, Nav1.1 is found at nodes of Ranvier of multiple tracts (Duflocq et al., 2008). Among peripheral nerves, Nav1.1 is found mainly in dorsal root ganglion somatosensory neurons (Fukuoka et al., 2008), possibly positioned to compensate in hibernators for any failure of normally more dominant Nav1.6–1.9 channels. While many of these paths are involved with proprioceptive feedback from muscles (Espino et al., 2022), mostly inactive in hibernation, there are others that have been associated with mechanical pain stimulation (Osteen et al., 2016), which could be vital to the hibernating animal. Thus, Nav1.1 and 1.2, given the M-for-L substitution in the sequence NQATL, could hypothetically serve to avoid slow inactivation at low temperature in specific neural pathways, but, unlike cardiac cells, they do not seem to have opportunities to do so together.

### M-for-L mutation in DIS6 and inactivation

Various alternatives to the classic chain-and-plug model of fast inactivation are under consideration, mostly involving some form of conformational change that would close down the channel, presumably near the cytoplasmic end, rather than simply plugging it (Pan et al., 2018; Hull and Lampert, 2025). As for slow inactivation, a model of a slow inactivated channel (Chen et al., 2023) implicates the same region of the S6 segments and likely the same eight residues highlighted in blue in Fig. 1. The asparagine, DIS6N, is a near neighbor and can potentially clash with one of those eight residues, namely L802 in adjacent DIIS6, so that an influence on the closing of that bottleneck is plausible. Indeed, Wang and Wang (1997) found that substituting alanine for DIS6N of rat Nav1.4 (N434A) made both fast and slow inactivation more rapidly developing. Given those results, one can only surmise that the hydrogen bonds predicted between that highly conserved asparagine and the neighboring methionine replacing leucine (Fig. 2 B) would hinder the repositioning of the asparagine during any conformational changes and would retard inactivation, fast or slow.

Such weak attractions would likely be insignificant at normal mammalian body temperature, but as cells are cooled and approach 0°C, those bonds would become stronger. They would then create a more significant energy barrier (or “energy of activation”) for the movement of the affected side chains and for the movements of the whole molecules relative to each other, making those movements acutely sensitive to the lowered temperature.

### Conclusion

Avoidance of slow inactivation and possibly delay of fast inactivation of still operative v.g. channels helps create large (“giant”) AP at 6°C or lower in atria of hibernating ground squirrels. TTX-sensitive v.g. Na channels, Nav1.1–1.4 and Nav1.6, are found in atria in addition to the principal v.g. Na channel, Nav1.5, and appear to be required for normal function. They may also support or replace the activity of Nav1.5 under stringent circumstances, like profoundly low body temperature. Mutation of the highly conserved single asparagine in DIS6 of those TTX-sensitive channels shortens the time for onset of fast inactivation and accelerates the use-dependent development of slow inactivation. Adjacent to that asparagine, an M-for-L mutation occurs at least two of four TTX-sensitive channels in most hibernating mammals, but not in those of nonhibernators. Among diverse species of hibernators, the mutation is pervasive in Nav1.1 and 1.2 and sporadic in Nav1.3 and 1.4. The persistent recurrence of this mutation both among species and among genes and exclusively in hibernators argues its importance for cold adaptation.

## Data Availability

No new data were generated or analyzed in support of this study. The data underlying Table 2 are openly available in the NCBI Protein database at https://www.ncbi.nlm.nih.gov/protein. Accession numbers used for individual species are provided in Table 1. Data underlying Figs. 1 and 2 are based on a molecular model available for download from the RCSB Protein Data Bank at https://www.rcsb.org/.
