# Correction: Evolution, hibernation, and inactivation of voltage-gated Na channels

**DOI:** 10.1085/jgp.20251392308102026c

**Published:** 2026-08-31

**Authors:** John S. Willis

Vol. 158, No. 5 | https://doi.org/10.1085/jgp.202513923 | July 7, 2026.

The author regrets that, during final file preparation, the last row of Table 1 providing NCBI accession numbers for human data was mistakenly removed. Additionally, the accession numbers in the SCN2A column for hedgehog, guinea pig, and deer mouse have been updated. In the SCN3A column, the accession number for elephant has been deleted and replaced by “N.A.”

**Table 1.** NCBI Accession numbers for entries in Table 2

​	SCN1A	SCN2A	SCN3A	SCN4A	SCN8A
13-Lined ground squirrel	XP_077873649	KAG3274613	N.A.	XP_021584085	XP_077870796
Arctic ground squirrel	XP_077654796	XP_026266882	XM_026411111	KAM4822770	KAM4840914
Yellow-bellied marmot	XP_027801819	XP_027801838	N.A.	XP_027802102	XP_027805589
Groundhog	XP_058436131	XP_058436122	XP_046303543	XP_046283698	XP_058432357
European marmot	XP_015334753	XP_015334460	N.A.	XP_015360302	XP_048658711
Golden hamster	XP_040601424	XP_021088572	N.A.	XP_005070068	XP_040609720
European hedgehog	XP_060033761	**XP_060033766**	N.A.	XP_060058537	XP_060051330
Little brown bat	XP_023601115	XP_036176358	XP_023601169	XP_023612521	XP_023615422
Big brown bat	XP_054578784	XP_054578791	N.A.	XP_008147646	XP_054575045
Beaver	N.A.	XP_020007198	XP_073927097	XP_073901513	XP_073939522
Guinea pig	XP_063115272	**XM_063259190**	XP_023416928	XP_063098664	XP_063110857
Deer mouse	XP_076425700	**XP_052581787**	XP_076425693	XP_015849809	XP_076412978
Norway rat	NP_110502	NP_036779	NP_001424699	NP_037310.2	XP_063119207
Eastern gray squirrel	XP_047400384	XP_047399883	XP_047400815	XP_047400804	XP_047404261
Rabbit	N.A.	XP_008256916	XP_069926841	XP_002719526	XP_051701456
Dog	XP_038302870	XP_013966299	XP_038302855	XP_038531929	XP_038294064
Shrew	XP_054975660	XP_054978862	XP_004601169	XP_054987535	XP_055976251
Elephant	XP_064143113	XP_003405869	**N.A.**	XP_064126987	XP_023412327
Horse	XP_070098085	XP_023478830	XP_070098078	NP_001075230	XP_070127551
**Human**	**P35498**	**M94055**	**AAK00219.1**	**P35499**	**AAD15789**

In Table 2, in the European hedgehog row of the SCN2A Nav1.2A column, the mutation has been corrected to “L.” Also, the technical name for deer mouse, “*Peromyscus maniculatus*,” has been changed to “*Peromyscus* spp.” For the Norway rat Nav1.3A, “L” has been entered.

**Table 2.** Prevalence of methionine-for-leucine substitution in TTX-sensitive v.g. Na channels of the hearts of hibernators

Common name	Technical name	NQATX.., X = ?					
		SCN1A Nav1.1A	SCN2A Nav1.2A	SCN3A Nav1.3A	SCN8A Nav1.6A	SCN4A Nav1.4A	Hibernator?
13-Lined ground squirrel	*Ictidomys tridecemlineatus*	M	M	______	L	L	Yes
Arctic ground squirrel	*Urocitellus parryii*	M	M	L	L	L	Yes
Yellow-bellied marmot	*Marmota flaviventris*	M	M	______	L	L	Yes
Woodchuck or groundhog	*Marmota monax*	M	L	M	L	L	Yes
European marmot	*Marmota marmota*	M	L	______	L	L	Yes
Golden hamster	*Mesocricetus auratus*	L	L	______	L	L	Yes
European hedgehog	*Erinaceus europeus*	M	**L**	L	L	L	Yes
Little brown bat	*Myotis lucifugus* or *myotis*	M	M	L	L	M	Yes
Big brown bat	*Eptesicus fuscus*	M	M	______	L	M	Yes
Beaver	*Castor canadensis*	_____	L	L	L	L	No
Guinea pig	*Cavia porcellus*	L	L	______	L	L	No
Deer mouse	***Peromyscus* spp.**	L	L	______	L	L	No
Norway rat	*Rattus norvegicus*	L	L	L	L	L	No
E gray squirrel	*Sciurus carolinensis*	L	L	______	L	L	No
Rabbit	*Oryctolagus cuniculus*	______	L	______	L	L	No
Dog	*Canis lupus familiaris*	L	L	______	L	L	No
Shrew	*Sorex araneus*	L	L	______	L	L	No
Elephant	*Loxodonta africanus*	L	L	______	L	L	No
Horse	*Equus caballus*	L	L	L	L	L	No
Human	*Homo sapiens*	L	L	______	L	L	No

The segment in which an M-for-L mutation is found is in the sixth transmembrane segment of domain one (DIS6) and begins NQAT in most Nav1s. (In Nav1.4, glutamate replaces glutamine, thus NEAT). Criteria for the selection of hibernators are provided in Materials and methods. Briefly, nonhibernators resist lowering of body temperature below 30°C in a cold environment by increasing heat production and insulation. Hibernators listed in the table in low ambient temperature and under appropriate circumstances, specific to the species, allow or even promote a decrease in core temperature to below 10°C and to as low as 0°C and regulate temperature and respiration at that low temperature for days or weeks. Dashed lines indicate that no sequence was available in the NCBI database. In the headings of each column, gene names are shown above protein identities.

The corrected tables are provided with the updated information in bold. The errors remain only in PDFs downloaded before August 28, 2026.

